# Radiation-Induced Synthesis and Superparamagnetic Properties of Ferrite Fe_3_O_4_ Nanoparticles

**DOI:** 10.3390/nano14121015

**Published:** 2024-06-12

**Authors:** Amel Zorai, Abdelhafid Souici, Daniel Adjei, Diana Dragoe, Eric Rivière, Salim Ouhenia, Mehran Mostafavi, Jacqueline Belloni

**Affiliations:** 1 Laboratoire de Physico-Chimie des Matériaux et Catalyse, Faculté des Sciences Exactes, Université de Bejaia, Bejaia 06000, Algeria; abdelhafid.souici@univ-bejaia.dz (A.S.); salim.ouhenia@univ-bejaia.dz (S.O.); 2 Institut de Chimie Physique, UMR 8000 CNRS, Université Paris-Saclay, Bâtiment 349, 91405 Orsay, France; daniel.adjei@universite-paris-saclay.fr (D.A.); mehran.mostafavi@universite-paris-saclay.fr (M.M.); 3 Laboratory for Vascular Translational Science, UMR 1148 INSERM, Université Sorbonne Paris Nord, Université Paris Cité, 93000 Bobigny, France; 4 Institut de Chimie Moléculaire et des Matériaux d’Orsay, UMR 8182 CNRS, Université Paris-Saclay, Bâtiment Henri Moissan, 19 Avenue des Sciences, 91400 Orsay, France; diana.dragoe@universite-paris-saclay.fr (D.D.); eric.riviere@universite-paris-saclay.fr (E.R.)

**Keywords:** Fe_3_O_4_ nanoparticles, pulse radiolysis, gamma radiolysis, coprecipitation, superparamagnetic properties

## Abstract

Ultra-small magnetic Fe_3_O_4_ nanoparticles are successfully synthesized in basic solutions by using the radiolytic method of the partial reduction in Fe^III^ in the presence of poly-acrylate (PA), or by using the coprecipitation method of Fe^III^ and Fe^II^ salts in the presence of PA. The optical, structural, and magnetic properties of the nanoparticles were examined using UV–Vis absorption spectroscopy, high-resolution transmission electron microscopy (HRTEM), X-ray diffraction (XRD), and SQUID magnetization measurements. The HRTEM and XRD analysis confirmed the formation of ultra-small magnetite nanoparticles in a spinel structure, with a smaller size for radiation-induced particles coated by PA (5.2 nm) than for coprecipitated PA-coated nanoparticles (11 nm). From magnetization measurements, it is shown that the nanoparticles are superparamagnetic at room temperature. The magnetization saturation value *Ms* = 50.1 A m^2^ kg^−1^ of radiation-induced nanoparticles at 60 kGy is higher than *Ms* = 18.2 A m^2^ kg^−1^ for coprecipitated nanoparticles. Both values are compared with nanoparticles coated with other stabilizers in the literature.

## 1. Introduction

In recent years, magnetic nanoparticles have received increasing attention because of their physical and chemical properties that are required for a wide range of technological applications, principally in magnetic storage technology [1], catalysis [2], and nanomedicine [3,4,5].

Among these nanoparticles, the ferrites MFe_2_O_4_ (M = Fe, Co, Mg, …) of ultra-small size may fulfill the features required for the miniaturization of the processes and, in the case of non-toxic Fe_3_O_4_, for functionalization via biocompatible molecules in medical treatments. Moreover, they may exhibit superparamagnetic behavior. This implies that the upper limit of temperature where the magnetization hysteresis disappears (or blocking temperature *T_B_*) is equal to or lower than 300 K [6].

The synthesis of the MFe_2_O_4_ ferrites has been extensively explored using different methods including co-precipitation [7,8,9], sol–gel [10], solvothermal [11], or hydrothermal synthesis [12], thermal decomposition [13,14], reduction in boiling polyol [15], or reduction via alkalide in ethers [16].

The radiolytic method is a promising alternative for the synthesis of magnetic nanoparticles [17]. This process does not require the presence of a chemical reductant because the irradiated solvent supplies the reducing species. Moreover, ionizing radiation has a high penetrating power. Hence, reducing radicals are produced as close as possible to the ions, unlike in chemical methods that involve reagents mixing and diffusion, with a high dependence on the agitation and volumes involved. As in the synthesis of metal or semiconductor nanoparticles [18,19,20], radiolysis also enables the bottom-up processes of particle nucleation and growth to be controlled by modulating the irradiation dose rate. The choice of the stabilizer is also crucial. For example, polyacrylate ions can restrict the size of Ag [21], Cu [22], or Pt oligomers [23] to only a few atoms. These features explain why the radiolytic route leads to the production of very small nanoparticles with a homogeneous distribution. In addition, pulse radiolysis offers the possibility of studying the different growth steps of metal and semiconductor clusters in real time [24,25].

Recent studies have demonstrated that superparamagnetic iron ferrite Fe_3_O_4_ nanoparticles [26,27] and cobalt ferrite CoFe_2_O_4_ nanoparticles [28] can be synthesized using radiolysis.

In this work, we focus our investigations on the formation of ultra-small iron magnetite nanoparticles and their superparamagnetic properties using the partial radiolytic reduction method of the ferric hydroxide complex without exploiting the homogeneous penetration properties of radiation. In view of possible biomedical applications [29], the particles are coated with poly(acrylate) ions (PA), which are biocompatible and have been previously shown to be the most efficient stabilizer [21,22,23]. The structural and magnetic properties of these radiation-induced and PA-coated nanoparticles are compared with those of particles coated with Arabic gum (AG) or synthesized using the method of coprecipitation of ferric and ferrous hydroxides, also coated with PA. Both methods avoid contamination by using the products of chemical-reducing agents and are performed at room temperature, where the sintering of the particles is inhibited.

## 2. Materials and Methods

All high-grade reagents were obtained from Sigma–Aldrich Chemistry (St. Quentin Fallavier, France). Water (Milli-Q, Merck KGaA, Darmstadt, Germany) with a resistivity of 18 MΩ cm was used in all experiments.

### 2.1. Coprecipitaion Synthesis of Fe_3_O_4_ Nanoparticles

Aqueous solutions of ferric chloride hexahydrate FeCl_3_, 6H_2_O, ferrous chloride tetrahydrate FeCl_2_, and 4H_2_O were mixed. The salt of FeCl_2_, 4H_2_O is initially slightly more concentrated than the required half part of the final FeCl_3_, the 6H_2_O concentration ([FeCl_3_, 6H_2_O] = 0.06 mol L^−1^), because the coprecipitation is carried out in the presence of air and Fe^II^ is somewhat oxidized in solution. Preliminary assays indicated that the optimal concentration corresponds to [FeCl_2_, 4H_2_O] = 0.1 mol L^−1^. For this comparison, the stabilizer added to the mixture was the same as in radiolytic synthesis. The polyacrylate ions (PAs) (final concentration 0.1–0.5 mol L^−1^) inhibit further growth of magnetite particles during their formation. In the last step, the pH of the solution was constantly monitored, as the NH_4_OH molar solution was added dropwise under vigorous magnetic stirring up to a pH of 12. The color changes rapidly from orange to black, and Fe_3_O_4_ particles are formed. The higher the PA concentration, the longer the time evolved before the nanoparticles fall to the bottom and the supernatant becomes clear (Figure S1). Note that the entire coprecipitation synthesis is carried out in the presence of air, in contrast with the irradiation method, where the ferrous ions are protected from O_2_ up to the formation of stable Fe_3_O_4_ nanoparticles.

### 2.2. Irradiation Synthesis

For the gamma-radiolysis experiments and sample characterization, aqueous solutions containing iron (III) chloride hexahydrate (FeCl_3_, 6H_2_O) at 10^−2^ mol L^−1^ were used. The solutions were mixed with 10^−1^ mol L^−1^ isopropanol ((CH_3_)_2_CHOH) as an OH^•^ radical scavenger, and 3 × 10^−3^ mol L^−1^ polyacrylate ions (PA) as stabilizers. Some experiments have been also performed using the Arabic gum (AG) stabilizer. The pH of the solution was constantly monitored as the NH_4_OH molar solution was added dropwise under vigorous magnetic stirring up to a pH of 12. Finally, the solution was deaerated through flushing with N_2_ (Alpha Gaz, Air Liquide, Paris, France) through a flask septum before irradiation. The color changed rapidly from orange to black with irradiation. Then, tiny black particles of Fe_3_O_4_ precipitated and the supernatant became transparent.

To measure the UV–vis absorbance of the solutions at an increasing cumulative gamma dose, a special glass device was used with a Pyrex^®^ part for irradiation, connected to another silica optical cell for spectrophotometric measurements. The solutions were 10-times diluted. Before irradiation, the samples were thoroughly deaerated with N_2_.

The samples were then exposed to a panoramic ^60^Co γ-ray source (Commissariat à l’Énergie Atomique, Saclay, France). During solution irradiation, the energy is absorbed by the most abundant water molecules (Reaction (1)), and the following species are formed:

(1) $$H_{2}O\overset{\gamma \;irradiation}{\to }e_{aq}^{-}(2.8),\;H^{+}(2.8),\;H^{•}\;(0.62),\;H_{2}\;(0.47),\;{OH}^{•}\;\left(2.8),\;H_{2}O_{2}\;(0.73\right)$$

(in brackets are the yields at pH 7 in 10^−7^ mole J^−1^ units) [30].

The dose rate of 2.3 kGy h^−1^ was measured using the Fricke dosimeter, based on the oxidation of ferro cyanide ions under acidic and aerated conditions, with *G*(Fe^3+^) = 16.2 × 10^−7^ mol J^−1^.

In a basic medium, the $H^{•}$ radicals give rise to solvated electrons:

(2) $$H^{•}+OH^{-}\to e_{aq}^{-}\;\;\;({pK}_{a}=\;9.6)$$

Hydrogen peroxide is in the form of the anion $HO_{2}^{-}$ [31], and isopropanol is in the form of the anion isopropanolate ${\left(CH_{3}\right)}_{2}CHO^{-}$.

The radicals $OH^{•}$ are scavenged by isopropanolate, and reducing radicals ${\left(CH_{3}\right)}_{2}CO^{-}$ are formed:

(3) $$OH^{•}+{\left(CH_{3}\right)}_{2}CHO^{-}\to {\left(CH_{3}\right)}_{2}C^{•}O^{-}+H_{2}O$$

At pH 12, the trivalent ferric ions are complexed as ${Fe}^{III}{\left(OH\right)}_{4}^{-}$ [32], and the divalent ferrous ions are complexed as ${Fe}^{II}{\left(OH\right)}_{3}^{-}$ [33].

Under irradiation, both agents, the hydrated electrons $(e_{aq}^{-})\;$ and ${\left(CH_{3}\right)}_{2}C^{•}O^{-}\;$ radicals, are homogeneously distributed throughout the solution, and their action is strongly reductive at room temperature. The ${Fe}^{III}{\left(OH\right)}_{4}^{-}$ ions are progressively reduced to ${Fe}^{II}{\left(OH\right)}_{3}^{-}\;$ ions by up to one-third of the initial amount:

(4) $$e_{aq}^{-}+{Fe}^{III}{\left(OH\right)}_{4}^{-}\to {Fe}^{II}{\left(OH\right)}_{3}^{-}+{OH}^{-}$$

(5) $${\left(CH_{3}\right)}_{2}C^{•}O^{-}+{Fe}^{III}{\left(OH\right)}_{4}^{-}\to {Fe}^{II}{\left(OH\right)}_{3}^{-}+{\left(CH_{3}\right)}_{2}CO+{OH}^{-}$$

Then, as soon as the ratio of Fe^II^/Fe^III^ = ½ is reached in the mixed ferrous and ferric hydroxide complexes, a precipitate of the black magnetite nanoparticles of Fe_3_O_4_ surrounded by the stabilizer is formed.

For the pulse radiolysis experiments, aqueous solutions contained 0, 2, or 5 × 10^−4^ mol L^−1^ complexed ferric ions ${Fe}^{III}{\left(OH\right)}_{4}^{-}$ at pH 12 (NaOH). The optical cell was connected to a reservoir using a circulating pump and the solution was thoroughly deaerated through nitrogen flushing. It was then submitted in the optical cell to a picosecond pulse provided by the electron accelerator ELYSE (8 MeV, 5 ps) (Laboratoire de l’Accélérateur Linéaire, Orsay, France) [34]. The spectrophotometric probe beam was collinear to the electron beam. The transient absorbance was measured using a repetitive flash lamp and a streak camera Hamamatsu [35]. The dose per pulse (approximately 10 Gy) was measured in neat water from the absorbance of $e_{aq}^{-}$ at 380–750 nm before each series of experiments, considering the initial yield of the solvated electrons to be *G*($e_{aq}^{-}$)_10ps_ = 4.4 × 10^−7^ mol J^−1^ [35].

### 2.3. Characterization of Ultra-Small Magnetite Nanoparticles

In the presence of a magnet, the black nanoparticles, produced by irradiation or coprecipitation, are attracted to the walls of the vessel, confirming their magnetic properties. However, this collection requires longer times when the PA concentration increases, indicating that the particles are smaller (Figure S1). UV-visible spectra of the irradiated solutions were recorded using a Hewlett-Packard 8453A spectrophotometer (Hewlett Packard Inc., Puteaux, France).

To investigate the structural and magnetic properties of the particles for both syntheses (using radiolysis or coprecipitation), the particles were separated from the supernatant via centrifugation, and were washed with ethanol and deionized water. Thus, the nanoparticles are reversibly suspended in liquid. The rinsing cycle was repeated twice before the final centrifugation and drying. Despite the repeated washing cycles, it is not excluded that the samples yet contained adsorbed PA molecules and that they were not bare when weighed and characterized. The nanoparticles synthesized at low doses were promptly dried directly after the centrifugation to prevent the oxidation of orange ferric oxides during washing.

The nanoparticle morphology and size distribution were investigated using high-resolution transmission electron microscopy (HRTEM) and selected area electron diffraction (SAED) using a JEOL 2100 Plus instrument (Jeol, Tokyo, Japan) operating at 200 kV fitted with a Gatan Rio 16 camera. 

The structure of the iron oxide nanoparticles was analyzed using X-ray diffraction (XRD) using a Panalytical X’Pert Pro MPD diffractometer (Malvern Panalytical, Malvern, UK) with Cu Kα radiation (λ = 1.54058 Å), followed by Rietveld refinement [36].

XPS measurements were performed on a K Alpha spectrometer (Thermofisher Scientific, Waltham, MA, USA), equipped with a monochromated X-ray Source (Al K_α_, 1486.7 eV) with a spot size of 400 µm, corresponding to an irradiated area of ~1 mm^2^. The hemispherical analyzer was operated in constant analyzer energy mode (CAE), with a pass energy of 200 eV and a step of 1 eV for the acquisition of surveys spectra, as well as pass energies of 50 eV and 20 eV with a step of 0.1 eV for the acquisition of narrow spectra. A “dual beam” flood gun was used to neutralize the charge build-up. The binding energies were calibrated against the neutral C1s component, with the binding energy set at 285.0 eV. The uncertainty of the analyzer binding energy was ±0.2 eV. The samples were mounted on a sample holder using aluminum foil masks. 

The spectra were treated by means of CasaXPS software (Version 2.3.25) [37]. The fitting procedure implied the use of Gauss–Lorentz lineshapes with 30% Lorentian character after the extraction of a Shirley-type background.

Magnetic measurements of the Fe_3_O_4_ nanoparticles were performed using a superconducting quantum interference device (SQUID) magnetometer (MPMS XL7 Quantum Design, San Diego, CA, USA) under a magnetic field of 50 Oe. After weighing, the samples were fixed with glue to avoid any orientation with respect to the magnetic field, and the specific magnetization value per unit weight was measured. Isothermal magnetization studies were performed by varying the applied field in the range of −50 kOe ≤ *H* ≤ 50 kOe, for temperatures of 5, 100, and 300 K. The specific feature of magnetization dependence on the field is the presence of hysteresis. The coercitive field, *Hc*, is the minimum field value required to obtain a change in the magnetization sign. The magnetization at saturation *M_s_* is the maximum value of *M*. Remanent magnetization *M_r_* is the value when the field decreases to zero. Zero-field cooling (ZFC) and field-cooling (FC) magnetization curves were measured at increasing temperatures with a constant magnetic field of 50 Oe and a temperature sweep rate of 2 K/min. When the magnetization is the same in both curves, the blocking temperature *T_B_* is reached, above which the material becomes superparamagnetic.

## 3. Results and Discussion

### 3.1. Pulse Radiolysis

**Figure 1:**
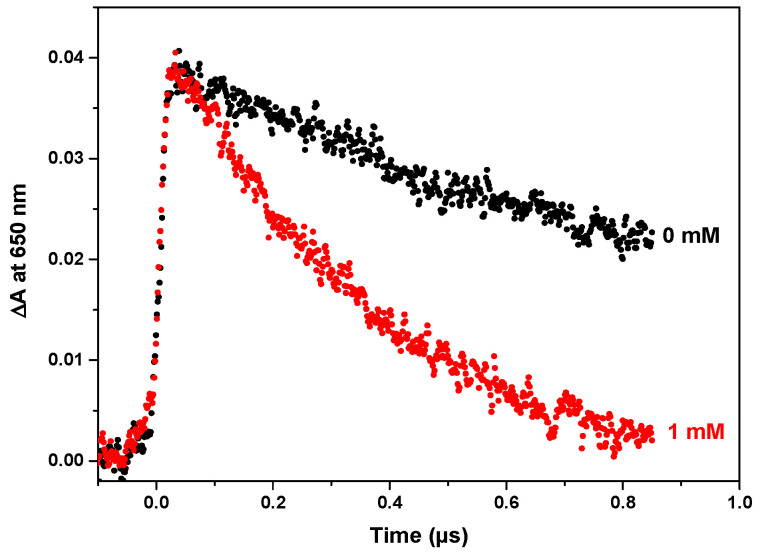
Time evolution of the differential absorbance at 650 nm of $e_{aq}^{-}\;$ in the presence or not of Fe^III^ at 10^−3^ mol L^−1^ and NaOH (pH~12). Dose: 10 Gy. Optical path: 5 mm.

In the pulse radiolysis study, we intend to observe the initial step of ferric ions reduction before further precipitation of Fe_3_O_4_. The Fe^III^ concentration in the solution is 10^−3^ mol L^−1^. The formation of Fe^II^ is restricted to the Fe^III^ reduction by the hydrated electrons scavenging. The time-resolved optical absorbance of $e_{aq}^{-}$ 650 nm after a pulse of 10 Gy is presented in Figure 1. The fast increase within a short pulse duration corresponds to the formation of the hydrated electron. The decay using Reaction (4) is of the pseudo-first-order.

Thus, the second-order rate constant is *k*_4_~10^9^ mol L^−1^ s^−1^. This value corresponds almost to a diffusion-controlled reduction reaction occurring between two negatively charged species: the hydrated electrons and the complexed ions ${Fe(OH)}_{4}^{-}$.

### 3.2. Optical Absorption Spectra of Gamma-Induced Solutions of Fe_3_O_4_ Nanoparticles

**Figure 2:**
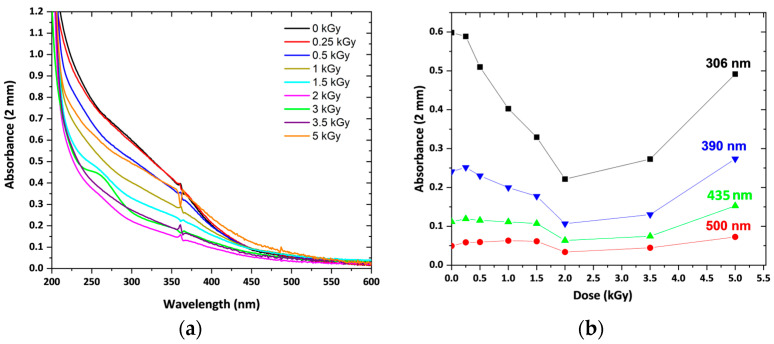
(**a**) Absorption spectra at an increasing cumulative dose of a solution containing 10^−3^ mol L^−1^ iron (III) chloride hexahydrate, 10^−3^ mol L^−1^ isopropanol, and 3 × 10^−4^ mol L^−1^ PA (pH 12). (**b**) Dose-dependent absorbance at 300, 390, 435, and 500 nm.

Before gamma irradiation, the solutions are orange and transparent. The pH conditions (pH = 12) are optimized in order to obtain the anionic complex of ferric hydroxide particles stabilized by PA (Figure 2) or AG (Figure S2). After gamma irradiation, not only are ${Fe(OH)}_{4}^{-}\;$ ions partly reduced to ${Fe}^{II}{\left(OH\right)}_{3}^{-}$ ions by hydrated electrons (Reaction (4)) and radicals of isopropanol (Reaction (5)), but magnetite Fe_3_O_4_ also precipitates.

The optical absorption spectrum before irradiation corresponds mostly to a rusty orange color of the ${Fe(OH)}_{4}^{-}\;$ ions and is superimposed with the ${Cl}^{-}$ ions’ absorbance and a weak band around 306 nm, specific to Fe^III^ (Figure 2a). Under irradiation, one-third of the ferric ions are progressively reduced in situ throughout the solution into ferrous ions by $e_{aq}^{-}\;and\;{\left(CH_{3}\right)}_{2}C^{•}O^{-}$ (Reactions (4) and (5)). The dose dependence of the absorbance at various wavelengths is shown in Figure 2b. Clearly, the absorbance decays at any wavelength up to 2 units. However, above 2 kGy, the absorbance increases after a minimum for wavelengths below 420 nm and decreases at wavelengths longer than 420 nm as an isosbestic point (Figure 2).

Above 6 kGy, the absorbance at any wavelength of the grey colloidal solution remains constant. Similar trends in the dose dependence of the absorbance are observed when the stabilizer is AG (Figure S2).

In addition to the reduction (Reactions (4) and (5)) of ${Fe(OH)}_{4}^{-}$ into ${Fe(OH)}_{3}^{-}$, a part of these ferrous ions is easily back-oxidized by the radiolytic hydrogen peroxide ($HO_{2}^{-}$ at pH 12) arising from Reaction (1) and having an oxidation yield of 1.5 × 10^−7^ mol J^−1^ (Reaction (6)). Because of the formation of the ferric species, ${Fe}^{III}OOH$ was observed as increasing with the dose [38]; therefore, we suggest that it is the product of the back oxidation of ${Fe(OH)}_{3}^{-}$ by $HO_{2}^{-}$ [31].

(6) $$2{Fe}^{II}{\left(OH\right)}_{3}^{-}\;+\;HO_{2}^{-}\;\to \;2{Fe}^{III}OOH\;+\;H_{2}O+3OH^{-}$$

Finally, the mixed complexed ferrous and ferric species ${Fe}^{II}{\left(OH\right)}_{3}^{-},$ 2${Fe}^{III}OOH$ loses further water molecules and precipitates into Fe_3_O_4_ black particles (Reaction (7)):

(7) $${Fe}^{II}{\left(OH\right)}_{3}^{-},\;2{Fe}^{III}OOH\;\to \;{Fe}_{3}O_{4}+{2H}_{2}O+OH^{-}$$

Meanwhile, the other part of the mixed ferrous and ferric hydroxo ions ${Fe}^{II}{\left(OH\right)}_{3}^{-}$, 2${Fe}^{III}{\left(OH\right)}_{4}^{-}$, which was not oxidized by hydrogen hydroxide, is precipitated after dehydration into black magnetite nanoparticles of Fe_3_O_4_ surrounded by the stabilizer.

(8) $${Fe}^{II}{\left(OH\right)}_{3}^{-},\;2{Fe}^{III}{\left(OH\right)}_{4}^{-}\;\to \;{Fe}_{3}O_{4}+{4H}_{2}O+3OH^{-}$$

**Figure 3:**
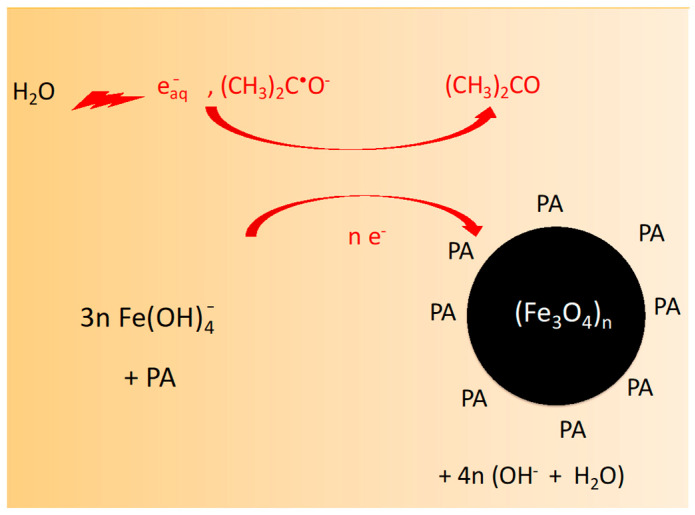
Scheme of the partial radiolytic reduction by $e_{aq}^{-}$ and ${\left(CH_{3}\right)}_{2}C^{•}O^{-}$ of ${Fe(OH)}_{4}^{-}$ ions into ${Fe}_{3}O_{4}$ nanoparticles at pH~12.

Overall, the one-step radiolytic reduction by $e_{aq}^{-}$ and ${\left(CH_{3}\right)}_{2}C^{•}O^{-}$ of ${Fe}^{III}{\left(OH\right)}_{4}^{-}$ ions into Fe_3_O_4_ nanoparticles is illustrated in the scheme of Figure 3.

As long as the dose of 6 kGy is not reached (or 60 kGy when the initial Fe^3+^ concentration is 10^−2^ mol L^−1^), the mixed ions are back oxidized when in contact with the air. This phenomenon has also been mentioned previously [38]. In contrast, the magnetite Fe_3_O_4_ nanoparticles are stable in the presence of oxygen. As a dose of 6 kGy is required before the particles are stable in air, we consider that the final formation of Fe_3_O_4_, corresponding to one-third of the initial concentration of ${Fe(OH)}_{4}^{-}$, occurs at this dose of 6 kGy.

The overall formation yield is equal to *G*(${Fe}_{3}O_{4}$) = *G*(Fe^II^) = 10^−3^/(3 × 6 × 10^3^) = 0.55 × 10^−7^ mole J^−1^. Even if we account for the transient oxidation by radiolytic hydrogen peroxide with 2*G*($H_{2}O_{2}$) = 1.46 × 10^−7^ mol J^−1^, the yield value is much lower than the reduction yield of the radiolytic species: *G(*$e_{aq}^{-}$) + *G*($H^{•}$) + *G*($OH^{•}$) − *G*($H_{2}O_{2}$) = 4. 7 × 10^−7^ mol J^−1^. In fact, during slow gamma irradiation, the part of the precipitated ferrite Fe_3_O_4_ particles is increased; moreover, because they also progressively adsorb most of the unreduced ferric ions (see Section 3.4), the remaining concentration of dissolved ions becomes very low, and they do not scavenge all the reducing species.

Note that the low formation yield of the cobalt ferrite CoFe_2_O_4_ [28] can also be explained in part by the back transient oxidation of Co^II^ into Co^III^ by $HO_{2}^{-}$.

The shape of the dose-dependent absorbance curves with a minimum of 2 kGy (Figure 2 and Figure S2) results from two trends, the first decreasing and the second increasing. The first decrease in absorbance at 306 nm, which is a specific wavelength of Fe^III^, is assigned to the reduction of Fe^III^ to Fe^II^ (Reactions (4)–(7)), partial re-oxidation into FeOOH, and Fe_3_O_4_ precipitation. If all molar absorption coefficients of these species were independent of the particle size, the absorbance variation would monotonously decrease without a minimum. Initially, the Fe_3_O_4_ particles are very small, and their scattering coefficient is much smaller than the differential absorption coefficient between ${Fe(OH)}_{4}^{-}$ or FeOOH and Fe_3_O_4_ (the absorbance of ${Fe}^{II}{\left(OH\right)}_{3}^{-}$ is negligible). The absorbance increase above 2 kGy is assigned to the increase in the molar scattering coefficient with the size of the growing Fe_3_O_4_ nanoparticles, or their aggregates.

### 3.3. XRD Structural Properties

#### 3.3.1. XRD Spectra of Coprecipitated Nanoparticles

**Figure 4:**
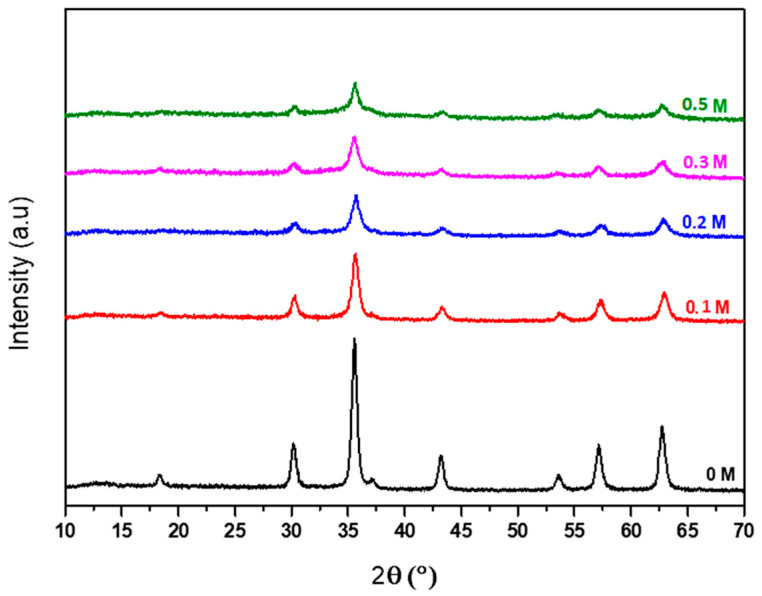
XRD spectra of coprecipitated Fe_3_O_4_ nanoparticles. From bottom to top: 0, 0.1, 0.2, 0.3, and 0.5 mol L^−1^ PA in a 10^−2^ mol L^−1^ Fe^III^ solution.

For comparison, the XRD spectra of particles obtained through coprecipitation are presented at various PA concentrations. Several intense XRD reflection peaks of the nanoparticles in the region of 2θ = 10–70° are shown in Figure 4.

The peaks correspond to (220), (311), (222), (400), (422), (511), and (440) planes of the cubic structure of the Fe_3_O_4_ spinel_._ The higher the PA concentration, the broader and less intense the peaks, indicating that the spinel crystallite size decreases. The average particle diameters calculated using Rietveld refinement are summarized in Table S1.

#### 3.3.2. XRD Spectra of Radiation-Induced Nanoparticles

**Figure 5:**
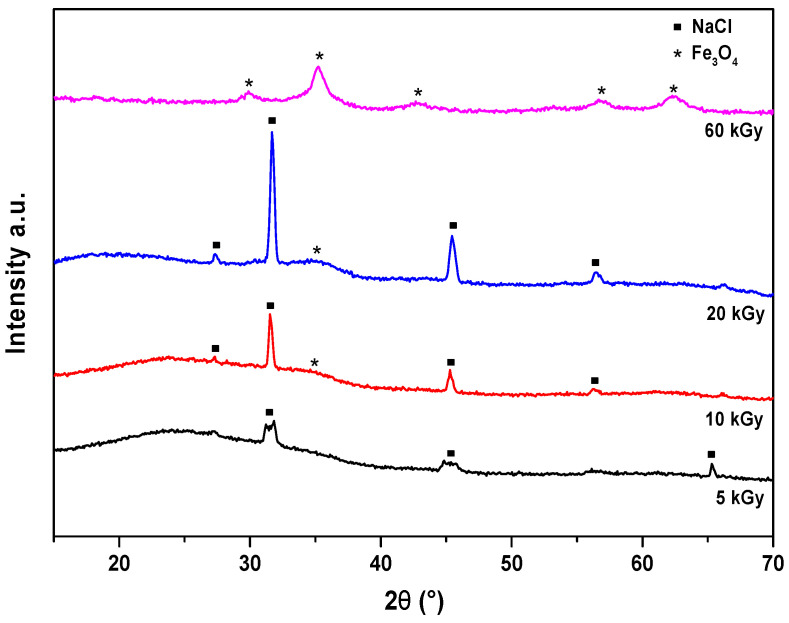
X-ray diffraction patterns of radiation-induced, PA-coated Fe_3_O_4_ nanoparticles formed at 5, 10, 20, and 60 kGy in a 10^−2^ mol L^−1^ Fe^III^ and 3 × 10^−3^ mol L^−1^ PA solution.

Several intense XRD reflection peaks of the radiation-induced nanoparticles synthesized in 10^−2^ mol L^−1^ solutions are observed in the region of 2θ = 15–70° (Figure 5). The crystallite sizes and morphologies were determined from the XRD data. Based on the Rietveld analysis technique, the entire pattern was fitted using combined analysis formalism [39] implemented in the MAUD (Version 2.9993) program [40]. A LaB_6_ standard powder from NIST was used to calibrate the instrumental contribution to the line broadening. Fourier analysis was used to deconvolve the instrumental and sample-broadening components of the measured XRD lines. Popa formalism was used to describe the crystallite sizes [41], and an arbitrary texture correction model was used to account for the moderately preferred orientations introduced in the Fe_3_O_4_ powder in a flat sample holder.

Samples irradiated at a dose of 5–20 kGy, which are particularly sensitive to oxidation by oxygen, were immediately dried. The diffraction spectra show only NaCl peaks and no other crystal peaks. This implies that the Fe_3_O_4_ samples are amorphous at this dose. For the sample irradiated at 10 kGy and more clearly at 20 kGy, the diffraction patterns show, in addition to NaCl, a weak signal at 35.2° of the magnetite spinel phase. This formation of magnetite, in addition to NaCl impurities, has already been observed for doses of 10, 14, and 20 kGy for the same precursor concentration of 10^−2^ M but with the stabilizer dextran at a dose rate of 26 kGy h^−1^ [42]. It has been reported that the dose, dose rate, and isopropanol concentration strongly influence the final product of gamma irradiation synthesis [43,44].

The diagram of the sample irradiated at 60 kGy and washed several times does not present any impurities and consists exclusively of a single phase corresponding to the spinel structure of Fe_3_O_4_ magnetite. To ensure that the magnetite is not oxidized to γ-Fe_2_O_3_ maghemite upon contact with oxygen, the lattice parameter was calculated as *a* = 8.399 Å, which corresponds fairly well to that of magnetite Fe_3_O_4_ (*a* = *b* = *c* = 8.404 Å) [40]. These results are, therefore, in agreement with dextran-coated particles [45], where the complete formation of Fe_3_O_4_ is also achieved at 60 kGy for the same initial Fe^III^ concentration. Note that when the initial Fe^III^ concentration is 10^−2^ mol L^−1^, which is ten times higher than for the optical studies above, the final dose required for obtaining stable Fe_3_O_4_ particles is now 60 kGy, and the yield is unchanged.

The XRD pattern at 60 kGy exhibits broad diffraction peaks with low intensity as for coprecipitated nanoparticles. On average, the diameter of the nanoparticles obtained at 60 kGy, calculated using Rietveld refinement, is 7.0 nm.

### 3.4. X-ray Photoelectron Spectroscopy Analysis of Radiation-Induced Nanoparticles

**Figure 6:**
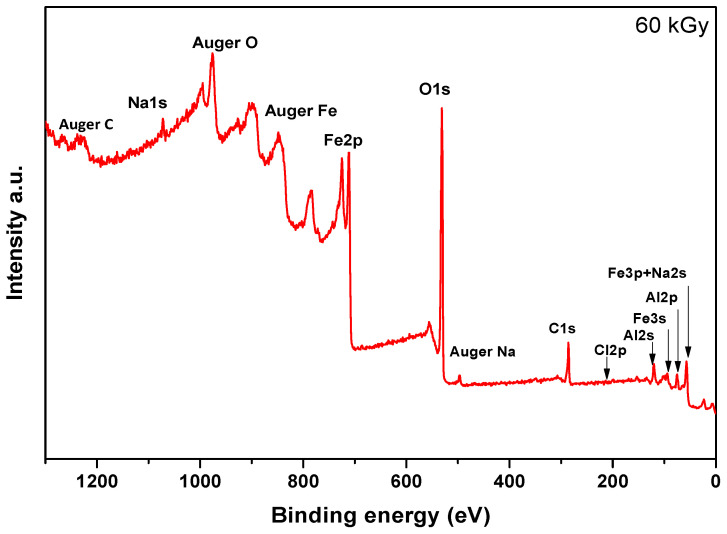
Survey XPS spectrum of PA-coated Fe_3_O_4_ nanoparticles obtained after 60 kGy in the presence of PA.

XPS measurements were conducted to reveal the surface information of radiation-induced ferrite nanoparticles. Figure 6 shows the survey spectra of the sample irradiated at 60 kGy. The presence of Fe and O is obvious, along with the carbon and Na pollution peaks and Al signals coming from the aluminum foil used to mount the samples.

**Figure 7:**
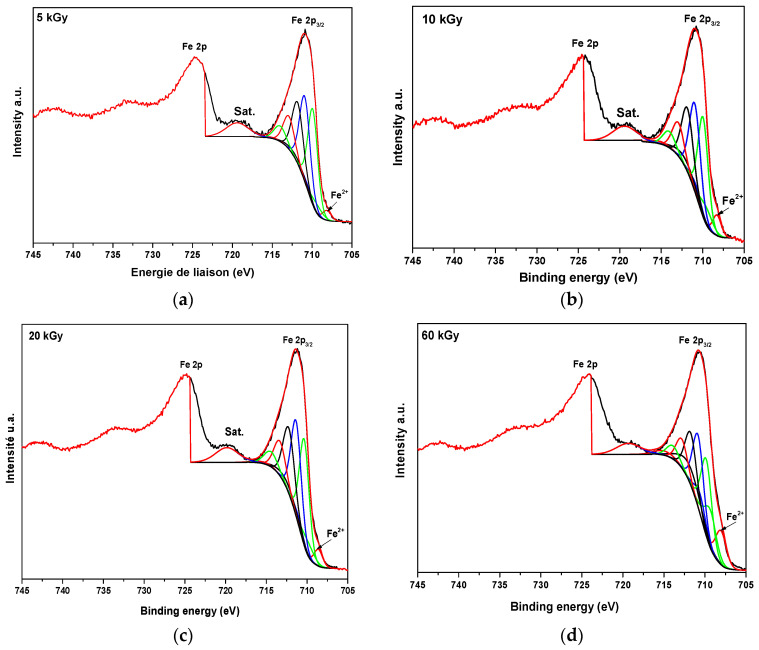
Decomposition of Fe2p core level spectra of the PA-coated Fe_3_O_4_ nanoparticles synthesized at (**a**) 5, (**b**) 10, (**c**) 20, and (**d**) 60 kGy in the presence of PA.

The core-level spectrum of iron consists of a doublet (Fe2p_3/2_ and Fe2p_1/2_), which results from the lifting of the spin-orbit coupling of the 2p levels (Figure 7). The spectrum is complex due to the presence of satellite structures and multiple effects of paramagnetic elements. A decomposition of Fe2p spectra corresponding to different irradiation doses was accomplished in order to evaluate the Fe^II^/Fe^III^ ratios and their evolution with the irradiation dose. Both Fe^II^ and Fe^III^ can be fitted using the Gupta and Sen multiplet structure derived from theoretical calculations [46]. The fitting parameters for both the multiplet and satellite peaks were taken from references [47,48]. Thus, five components (at the binding energies 708.4, 709.7, 710.9, 712.1, and 715.4 eV) were added to fit the Fe^II^, and six components (at 710.0, 711.0, 711.9, 713.0, 714.1, and 719.5 eV) were added corresponding to Fe^III^. The results for the areas of each peak are presented in Tables S2 and S3. The total areas of the peaks of Fe^II^ and Fe^III^ are summarized in Table 1.

The results of XPS analysis confirm the successful synthesis of PA-coated Fe_3_O_4_ nanoparticles at an irradiation dose of 60 kGy by obtaining a ratio Fe^3+^:Fe^2+^ = 2, confirmed also through XRD, which demonstrates the formation of crystalline Fe_3_O_4_ at 60 kGy. At lower doses, the Fe^III^/Fe^II^ ratio in particles does correspond to Fe_3_O_4_ with excess Fe^III^ at the surface. We conclude that Fe^III^ ions remained on the nanoparticles via adsorption and were separated with them from the supernatant during centrifugation.

**Figure 8:**
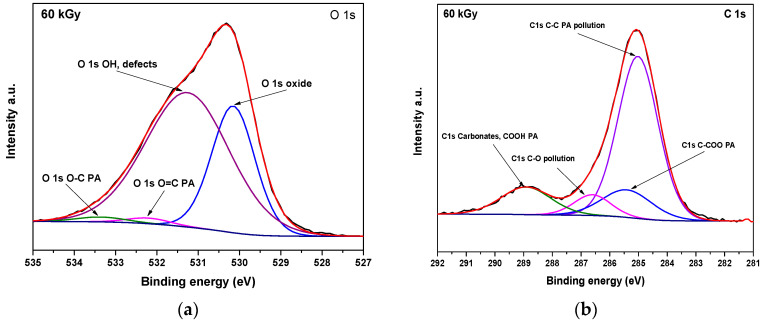
XPS spectra of radiation-induced iron oxide nanoparticles at 60 kGy: (**a**) O 1s; (**b**) C 1s.

The core-level spectra of O1s and C1s are presented in Figure 8a. Despite the fact that the contributions coming from PA are strongly interfered with, a successful fit could be performed that includes them. It is not possible, though, to unequivocally conclude the presence of PA coating from these results.

The two main components of O1s core-level spectra are located at 530.1 eV and attributed to the oxide and 531.3 eV, attributed to hydroxides and defects. Two small contributions at higher binding energy were added to account for PA; the one positioned at 532.2 eV was attributed to the double-bound O=C and the second, at 533.4 eV, to single-bounded O [49].

In a similar manner, besides the components coming from the pollution carbon, namely, C-C (285 eV), C-O (286.6 eV), and carbonates (288.9 eV), of the three other peaks representing the C-C (285 eV), C linked to carboxy function (285.4 eV) and C from carboxyl (288.9 eV) was considered as coming from PA [49].

### 3.5. High-Resolution Transmission Electron Microscopy Imaging

#### 3.5.1. TEM of Coprecipitated Fe_3_O_4_ Nanoparticles

For comparison, the TEM images of coprecipitated PA-coated Fe_3_O_4_ nanoparticles are presented in Figure S3. For [PA] = 0.2 and 0.5 mol L^−1^, the morphology of all the nanoparticles is homogeneous and spherical in shape with an average diameter of 13 and 11 nm, respectively. The particle sizes obtained using TEM are quite consistent with the results obtained through X-ray diffraction (Table S1).

#### 3.5.2. HRTEM Imaging of Radiation-Induced Nanoparticles of Fe_3_O_4_

**Figure 9:**
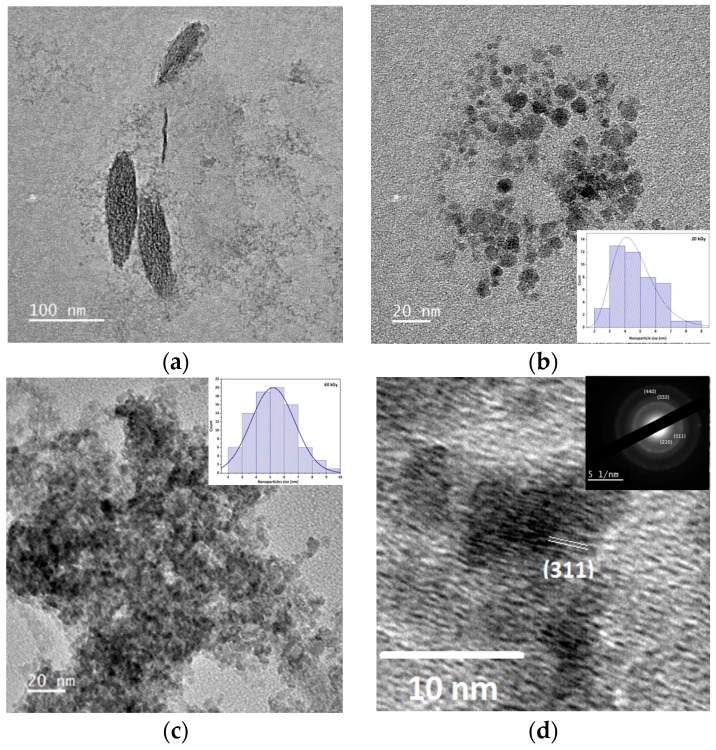
(**a**) TEM image after an irradiation dose of 20 kGy in the presence of PA showing long particles. (**b**) TEM image at 20 kGy in the presence of PA showing spherical particles. Inset: Lognormal size distribution of nanoparticles at 20 kGy, *D* = 4.0 nm. (**c**) TEM images of PA-coated Fe_3_O_4_ nanoparticles synthesized at 60 kGy. Inset: Lognormal size distribution of nanoparticles at 60 kGy, *D* = 5.2 nm. (**d**) HRTEM image of Fe_3_O_4_ nanoparticles of the (311) atomic plane. Inset: Electron diffraction pattern indexed with the crystallographic data of the Fd$\bar{3}$m spinel structure.

Figure 9 displays a TEM micrograph of Fe_3_O_4_ nanoparticles that were formed at an irradiation dose of 20 kGy. For nanoparticles synthesized at 20 kGy in the presence of PA, two types of morphology can be distinguished, including the spherical shape, which corresponds to Fe_3_O_4_ nanoparticles with a size of 5.2 nm (maximum of lognormal fit).

Some other particles with a more elongated shape are observed that could correspond to the presence of FeO(OH), as already detected using Mossbauer spectroscopy in the synthesis with microemulsion under gamma irradiation [50]. At this dose, only a partial reduction is observed.

After a complete reduction at 60 kGy with PA as a stabilizer, only Fe_3_O_4_ spherical nanoparticles are observed (Figure 9c and inset), which is consistent with the observations in the literature for the radiolytic synthesis of such systems [23]. The high-resolution image (Figure 9d) presents a region with, for example, a single particle of *D* = 5.2 nm. The image shows the high spinel crystallinity and structural homogeneity of the particle. These observations are in fair agreement with the XRD analysis.

The size is smaller than those observed for Fe_3_O_4_ nanoparticles also formed under irradiation at 30 kGy in the presence of PA (D~30 nm) [51] or synthesized through other methods [52]. Nevertheless, it is close to that observed for nanoparticles synthesized by radiolytic means in the presence of AG (Figure S4), dextran [53], or Triton-X [26]. It should be noted that, with the same stabilizer PA, the sizes of nanoparticles are much smaller (5.2 nm) (Figure 9) when synthesized using the radiolytic route than by coprecipitation (11 nm) (Figure S3), despite a much lower concentration of PA was used (3 × 10^−3^ instead of 0.5 mol L^−1^). In fact, the reduction using penetrating radiation is a more homogeneous nucleation process than the coprecipitation of ferrous and ferric ions, implying reactant mixing. In the synthesis of metal clusters, it was demonstrated that the radiation-induced crystal nuclei are more numerous and more easily protected from growth by the stabilizer; therefore, the final particle sizes are smaller than in a mixture of chemicals [18].

### 3.6. Magnetic Property

#### 3.6.1. Coprecipitated PA-Coated Fe_3_O_4_ Nanoparticles

**Figure 10:**
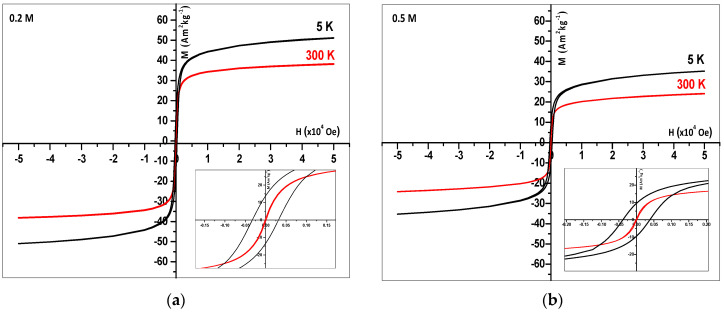
Hysteresis magnetization loops at T = 5 K and T = 300 K. (**a**) 0.2 and (**b**) 0.5 mol L^−1^ PA-coated ${Fe}_{3}O_{4}$ nanoparticles obtained via coprecipitation. Insets: Magnification of the low field region.

The magnetization curves, observed using SQUID for coprecipitated Fe_3_O_4_ nanoparticles coated by PA at 0.2 or 0.5 mol L^−1^, are presented in Figure 10. The magnetic parameters are given in Table 2. At 5 K, we observed a hysteresis of the magnetization variation. The coercitive field is *H_C_* = 400 Oe and the remanent magnetization is *M_r_* = 9.4 A m^2^ kg^−1^ at 0.5 mol L^−1^ PA (Table 2). However, at 300 K, the loop is almost closed and the hysteresis vanishes (*H_C_* = 4 Oe). In parallel, the remanent magnetization is only *M_r_* = 0.3 A m^2^ kg^−1^. We conclude that the behavior of coprecipitated nanoparticles becomes superparamagnetic at room temperature. The spins are contained in a monodomain, and their orientation follows immediately after the field changes.

In Figure 10, the magnetization values at saturation *M_S_* of the PA-coated Fe_3_O_4_ nanoparticles decrease as the temperature rises because of increasing thermal fluctuations. Note that the magnetization at saturation decreases when the PA concentration increases. Possibly, the nanoparticles are not bare, and a higher part of PA is still adsorbed in the presence of a higher concentration. Thus, the iron oxide weight would be lower than that of the sample taken for the magnetization calculation, being a lower limit. The measured *M_s_* values of Table 2 are lower than *M_s_* = 67 A m^2^ kg^−1^ [9] of the bare nanoparticles of the 25 nm synthesized via coprecipitation and electrohydraulic discharge treatment.

Figure S5 presents the field-cooled (*FC*) and zero-field-cooled (*ZFC*) magnetization curves of the PA-coated ${Fe}_{3}O_{4}$ nanoparticles measured at decreasing temperatures between 300 and 5 K with an applied magnetic field of *H* = 50 Oe. The *FC* magnetization decreases, and, in contrast, the ZFC magnetization increases at increasing temperatures.

As a reminder, the magnetic anisotropy energy at *T_B_* corresponds to the energy required for the spin reorientation of the magnetic nanoparticles that is very close to the thermal energy *k*_Boltzmann_ × *T*_B_. This property also indicates that the nanoparticles are small enough to constitute single magnetic domains (or monodomains) [54]. However, a broadening of the ZFC curve as in Figure 5 is consistent with the polydisperse nature of the magnetite nanoparticles, namely, with the associated distribution in particle size and individual anisotropy axes. The broadening is also likely due to dipolar interactions among particles in agglomerates. The broad maximum in Figure S5 indicates a contribution of the largest particles (Figure S3, insets) with interactions inside the agglomerates. They induce a shift in the blocking temperature beyond the measurement limit of 300 K. However, the absence of 300 K of hysteresis, even at large magnification, means that the great majority of nanoparticles are small enough to be superparamagnetic.

#### 3.6.2. Radiation-Induced Nanoparticles of PA-Coated Fe_3_O_4_

**Figure 11:**
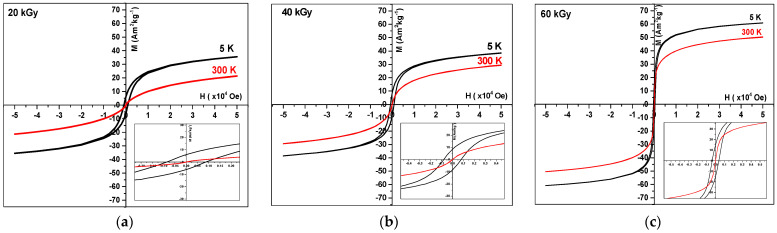
Magnetization hysteresis loops at 5 and 300 K of radiation-induced Fe_3_O_4_ nanoparticles coated by PA and synthesized at (**a**) 20, (**b**) 40, and (**c**) 60 kGy. Insets: Magnification of the low field region.

The magnetic properties of radiation-induced, PA-coated ${Fe}_{3}O_{4}$ nanoparticles investigated for 20, 40, and 60 kGy. The field-dependent magnetization was measured in the temperature range of 5–300 K and in magnetic fields ranging from *H* = −50 kOe to + 50 kOe (Figure 11).

The magnetization at saturation *M_s_* increases markedly with the dose used for the synthesis (Table 3). Moreover, at 5 K, the hysteresis loop is open for doses of 20, 40, and 60 kGy (black curves in Figure 11a–c, insets). In contrast, the loop is closed at T = 300 K for 20, 40 kGy, and 60 kGy (red curves in Figure 11, insets), suggesting that the nanoparticles are superparamagnetic at room temperature.

**Figure 12:**
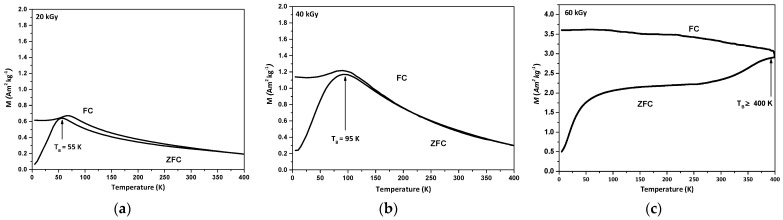
ZFC and FC magnetization curves of radiation-induced Fe_3_O_4_ magnetic nanoparticles coated by PA and synthesized at (**a**) 20, (**b**) 40, and (**c**) 60 kGy. (*H* = 50 Oe).

Figure 12 presents the temperature-dependence of the field-cooled (*FC*) magnetization curve at a fixed field of *H* = 50 Oe and the zero-field-cooled (*ZFC*) magnetization curve of the radiation-induced Fe_3_O_4_ nanoparticles coated by PA. The temperature of the maximum of the ZFC curve, corresponding to the blocking temperature *T_B_*, increases with the dose but is much lower than 300 K for 20 and 40 kGy. These results are in agreement with the absence of hysteresis in the loops of Figure 11a,b and confirm that the nanoparticles are superparamagnetic at room temperature.

For the nanoparticles synthesized at 60 kGy, the blocking temperature is *T_B_* ≥ 400 K (Figure 12c). However, as shown in the inset of Figure 11c at high magnification, the hysteresis loop is almost closed. This apparent disagreement between the loop and the blocking temperature has already been observed (Section 3.6.1) and for other coprecipitated ferrite nanoparticles [55]. The authors consider that the relaxation time of the *T_B_* measurement is too short compared to the particle relaxation time, and concluded from the closed loop that the nanoparticles were superparamagnetic. In addition, the temperature *T*_B_ of the particles is highly sensitive to their size distribution [56,57] and mutual interactions in agglomerates. The broad maximum in Figure S5 indicates a contribution of the largest particles (Figure S3, insets), possibly with interactions inside the agglomerates. They induce a shift in the blocking temperature to values higher than those for ultrasmall particles. However, the absence of 300 K of hysteresis, even at large magnification (Figure 11c, Inset), means that the great majority of nanoparticles are small enough to be superparamagnetic.

Noteworthy, the small size and the high magnetization value of the superparamagnetic radiation-induced Fe_3_O_4_ nanoparticles at 300 K are significant results in view of medical applications at room temperature.

When comparing both synthesis methods under our one-step conditions of coprecipitation and irradiation with the same PA-coating, the magnetization value at 300 K is much higher for radiation-induced Fe_3_O_4_ nanoparticles that are 5.2 nm in diameter (*M_S_* = 50.1 A m^2^ kg^−1^) (Table 3) than for coprecipitated nanoparticles of 13 nm and coated at 0.2 mol L^−1^ PA (*M_S_* = 38.5 A m^2^ kg^−1^) (Table 2). However, the diameter of the latter is larger than that of radiation-induced particles. The high potential of the radiolytic method for various applications is demonstrated.

Among radiation-induced Fe_3_O_4_ nanoparticles, the magnetization of those coated by PA is also higher (*M_S_* = 50.1 A m^2^ kg^−1^, *D* = 5.2 nm) than *M_S_* values for those coated by dextran sulfate (*M_S_*~39 A m^2^ kg^−1^, *D* = 6.2 nm) [27], or by Triton-X (35 A m^2^ kg^−1^, for 150 kGy, *D* = 5 nm) [26] (Table 4). Magnetization experiments have also been performed on radiation-induced nanoparticles coated by AG (Figures S6 and S7 and Table S4). However, the saturation magnetization at room temperature of these nanoparticles is only *M_S_* = 5.9 A m^2^ kg^−1^ (Table 4).

However, the magnetization of radiation-induced Fe_3_O_4_ nanoparticles (*M_S_* = 50.1 A m^2^ kg^−1^) is only slightly lower than that of CoFe_2_O_4_ nanoparticles (*M_S_* = 69.2 A m^2^ kg^−1^), in spite of their lower diameter (5.2 nm instead of 9 nm), as compared in Table 4 with the corresponding coatings.

## 4. Conclusions

Using the radiation-induced reduction of the Fe^III^ hydroxide complex ${Fe}^{II}{\left(OH\right)}_{4}^{-}$ in the presence of sodium polyacrylate or Arabic gum, or using the co-precipitation of Fe^III^ and Fe^II^ hydroxides in the presence of sodium polyacrylate, ferrite Fe_3_O_4_ nanoparticles were synthesized. The origin of the FeOOH oxide observed in the literature arises from the transient oxidation of part of ${Fe}^{II}{\left(OH\right)}_{3}^{-}$ by the radiolytic hydrogen peroxide.

The co-precipitation method and the radiolytic synthesis of iron ferrites were compared, both without any further treatment, using the same coating with the biocompatible and strong stabilizer PA. Ultra-small Fe_3_O_4_ nanoparticles were successfully synthesized in a single-crystalline phase with a relatively fair monodispersity. The XRD analysis and HRTEM images confirm their spinel structure with an average diameter of *D* = 13 nm for coprecipitated particles instead of only *D* = 5.2 nm for radiation-induced particles. The homogeneity of the nucleation-growth process controlled by PA is confirmed to be better during radiolysis than during coprecipitation. The hysteresis loop measurements at room temperature demonstrate their superparamagnetic character. However, the specific magnetization value at saturation is higher for the radiation-induced nanoparticles than for nanoparticles coprecipitated in this work, where non-magnetic adsorbed PA is still present. The stabilizer PA appears to be the most efficient stabilizer compared to others used in the literature for radiation-induced nanoparticles. The ultrasmall size combined with significant superparamagnetic properties at room temperature of the radiation-induced nanoparticles of Fe_3_O_4_ coated by a biocompatible stabilizer such as PA offers a very promising tool for medical applications in the hyperthermal therapy of cancer.

## Figures and Tables

**Table 1:** Relative areas of peaks associated with the fractions of surface Fe^II^ and Fe^III^ in nanoparticles coated with PA and synthesized at various doses.

Dose (kGy)	5	10	20	60
Fe^III^ Surface ions (%)	80.6	82.7	86.4	66.5
Fe^II^ Surface ions (%)	19.4	17.3	13.6	33.5

**Table 2:** Magnetic properties of PA-${Fe}_{3}O_{4}$ coprecipitated nanoparticles at 5 and 300 K.

	5 K			300 K		
[PA] (mol L^−1^)	*M_r_* (A m^2^ kg^−1^)	*M_S_* (A m^2^ kg^−1^)	*H_C_* (Oe)	*M_r_* (A m^2^ kg^−1^)	*M_S_* (A m^2^ kg^−1^)	*H_C_* (Oe)
0.2	13.7	51.1	300	0.5	38.5	12
0.5	9.4	35.2	400	0.3	24.2	4

**Table 3:** Magnetic properties at 5 and 300 K of radiation-induced Fe_3_O_4_ nanoparticles coated by PA and radiation-induced at 20, 40, and 60 kGy.

	5 K			300 K		
Dose (kGy)	*M_r_* (A m^2^ kg^−1^)	*M_S_* (A m^2^ kg^−1^)	*H_C_* (Oe)	*M_r_* (A m^2^ kg^−1^)	*M_S_* (A m^2^ kg^−1^)	*H_C_* (Oe)
20	5.9	35.5	870	0.08	21.4	23
40	8.5	38.7	780	0.18	29.5	21
60	14.8	61.3	390	0.23	50.1	5

**Table 4:** Comparison of diameters and magnetization values *M_s_* at 300 K for radiation-induced nanoparticles of Fe_3_O_4_ and CoFe_2_O_4_ coated by various stabilizers in the literature and in this work, together with coprecipitated nanoparticles coated by PA in this work.

Ferrite	Stabilizer	Synthesis	*D* (nm)	*M_S_* (300 K) (A m^2^ kg^−1^)	Refs.
Fe_3_O_4_	DEAE-dextran	γ-irradiation	6.4	39	Jurkin [53]
	PVA	γ-irradiation	2	11	Abedini [58]
	Triton-X	e-irradiation *	5	35	Dietrich [26]
	AG	γ-irradiation	5.6	5.9	This work
	PA	Coprecipitation	13	38.5	This work
	PA	γ-irradiation	5.2	50.1	This work
CoFe_2_O_4_	PVP	γ-irradiation	9	69.2	Zorai [28]

* Electron irradiation is a post-treatment following coprecipitation in microemulsion.

## Data Availability

Data are contained within the article and Supplementary Materials.

## Supplementary Materials

The following supporting information can be downloaded at: https://www.mdpi.com/article/10.3390/nano14121015/s1, Figure S1. Samples of Fe_3_O_4_ nanoparticles stabilized by PA at various concentrations, and observed 30 min after the coprecipitation. Figure S2. (a) Absorption spectra at different doses of a solution containing 10^−3^ mol L^−1^ iron (III) chloride hexahydrate, 10^−3^ mol L^−1^ isopropanol and 10^−3^ mol L^−1^ AG. (b) Absorbance as a function of dose at λ = 306 and 500 nm. Optical path: 2 mm. Figure S3. TEM images of Fe_3_O_4_ nanoparticles synthesized by coprecipitation and coated by PA at (a) 0.2 and (b) 0.5 mol L^−1^ and. Inserts: Size distribution of individual nanoparticles with lognormal fit. Figure S4. TEM images and diameter distribution with lognormal adjustment of Fe_3_O_4_ nanoparticles in the presence of arabic gum (AG), after an irradiation dose of: (a) 20 kGy; (b) 60 kGy. Figure S5. ZFC and FC magnetization curves for coprecipitated and PA-coated Fe_3_O_4_ nanoparticles at 0.2 and 0.5 mol L^−1^ (H = 50 Oe). Figure S6. Magnetization loops at 5 and 300 K for Fe_3_O_4_ AG-coated nanoparticles synthetized at 60 kGy. Inset: Zoom at low field values. Figure S7. ZFC curve (in black) and FC curve (in red) of the magnetization of nanoparticles Fe_3_O_4_ radiation-induced (dose = 60 kGy) and AG-coated, H = 50 Oe. Insert: In red: lognormal adjustment of the derivative d(ZFC − FC)/dT. Table S1. Rietveld parameters and phase analysis using the software MAUD (version 2.9993) for coprecipitated nanoparticles. Table S2. Relative areas of peaks associated to the fractions of surface Fe^II^ in nanoparticles coated by PA and synthesized at various doses. Table S3. Relative areas of peaks associated to the fractions of surface Fe^III^ in nanoparticles coated by PA and synthesized at various doses. Table S4. Magnetic properties of radiation-induced and AG-coated nanoparticles synthetized at 60 kGy.
