# Supplementary material for: Radiation-Induced Synthesis and Superparamagnetic Properties of Ferrite Fe3O4 Nanoparticles
Source: Nanomaterials (Basel). 2024 Jun 12;14(12):1015. doi: 10.3390/nano14121015 (PMC11206415; doi:10.3390/nano14121015)
Supplement: Supplementary file 1 [file nanomaterials-14-01015-s001.zip › nanomaterials-3015438-supplementary.pdf]

Supplementary Information

Radiation-Induced Synthesis and Superparamagnetic Properties of Ferrite Fe_3O_4 Nanoparticles

Amel Zorai [1,2,3,*], Abdelhafid Souici [1], Daniel Adjei [2], Diana Dragoe [4], Eric Rivière [4], Salim Ouhenia [1], Mehran Mostafavi [2] and Jacqueline Belloni [2,*]

1. Laboratoire de Physico-Chimie des Matériaux et Catalyse, Faculté des Sciences Exactes, Université de Bejaia, Bejaia 06000, Algeria; abdelhafid.souici@univ-bejaia.dz (A.S.); salim.ouhenia@univ-bejaia.dz (S.O.)
2. Institut de Chimie Physique, UMR 8000 CNRS, Université Paris-Saclay, Bâtiment 349, 91405 Orsay, France; daniel.adjei@universite-paris-saclay.fr (D.A.); mehran.mostafavi@universite-paris-saclay.fr (M.M.)
3. Laboratory for Vascular Translational Science, UMR 1148 INSERM, Université Sorbonne Paris Nord, Université Paris Cité, 93000 Bobigny, France
4. Institut de Chimie Moléculaire et des Matériaux d'Orsay, UMR 8182 CNRS, Université Paris-Saclay, Bâtiment Henri Moissan, 19 avenue des Sciences, 91400 Orsay, France; diana.dragoe@universite-paris-saclay.fr (D.D.); eric.riviere@universite-paris-saclay.fr (E.R.)

* Correspondence: amel.zorai@univ-paris13.fr (A.Z.); jacqueline.belloni@universite-paris-saclay.fr (J.B.)

2.1. *Coprecipitaion synthesis of Fe_3O_4 nanoparticles* stabilized by PA

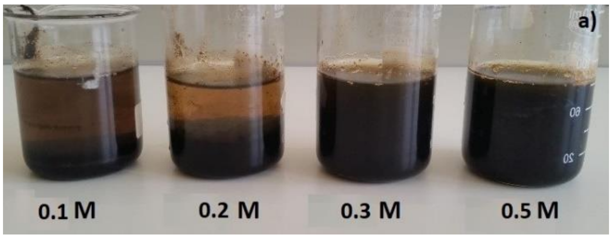

Figure S1. Samples of Fe_3O_4 nanoparticles stabilized by PA at various concentrations, and observed 30 minutes after the coprecipitation.

3.2. *Optical absorption spectra of gamma-induced solutions of Fe_3O_4 nanoparticles coated by AG*

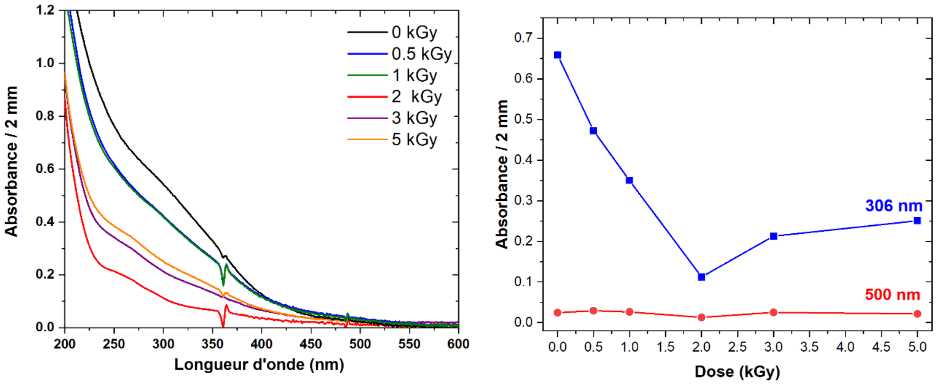

(a) (b)

Figure S2. a) Absorption spectra at different doses of a solution containing 10^{-3} mol L^{-1} iron (III) chloride hexahydrate, 10^{-3} mol L^{-1} isopropanol and 10-3 mol L^{-1} AG. b) Absorbance as a function of dose at λ = 306 and 500 nm. Optical path: 2 mm.

3.3. *XRD structural properties*

3.3.1. *XRD spectra of coprecipitated nanoparticles*

Table S1. Rietveld parameters and phase analysis using the software MAUD (Version 2.9993) for coprecipitated nanoparticles.

[PA] (mol L^{-1})	a = b = c (Å)	Diameter *D* (nm)
0	8.379	28.7
0.1	8.375	19.3
0.2	8.376	13.6
0.3	8.390	11.5
0.5	8.387	9.6

3.4. X-ray photoelectron spectroscopy analysis of radiation-induced nanoparticles

Table S2. Relative areas of peaks associated to the fractions of surface Fe^{II} in nanoparticles coated by PA and synthesized at various doses.

Peak BE (eV)	5 kGy	10 kGy	20 kGy	60 kGy
		**Fe^{II} % area**		
708.4	5.0	4.0	3.2	8.0
709.7	6.2	5.0	4.1	10.1
710.9	3.0	2.4	2.0	4.9
712.1	5.3	4.2	3.5	8.6
715.4	1.2	1.0	0.8	1.9
**Total Fe^{II} area%**	**20.7**	**16.6**	**13.6**	**33.5**

Table S3. Relative areas of peaks associated to the fractions of surface Fe^{III} in nanoparticles coated by PA and synthesized at various doses.

Peak BE (eV)	5 kGy	10 kGy	20 kGy	60 kGy
		**Fe^{III} % area**		
710.0	22.0	23.2	24.0	18.5
711.0	20.9	22	22.8	17.5
711.9	16.0	16.9	17.5	13.5
713.0	8.8	9.2	9.5	7.3
714.1	4.4	4.6	4.8	3.7
719.5	7.2	7.5	7.8	6
**Total Fe^{III} area%**	**79.3**	**83.4**	**86.4**	**66.5**

3.5. Transmission electron microscopy imaging of Fe_3O_4 nanoparticles

3.5.1. Transmission electron microscopy imaging of coprecipitated and PA-coated Fe_3O_4 nanoparticles

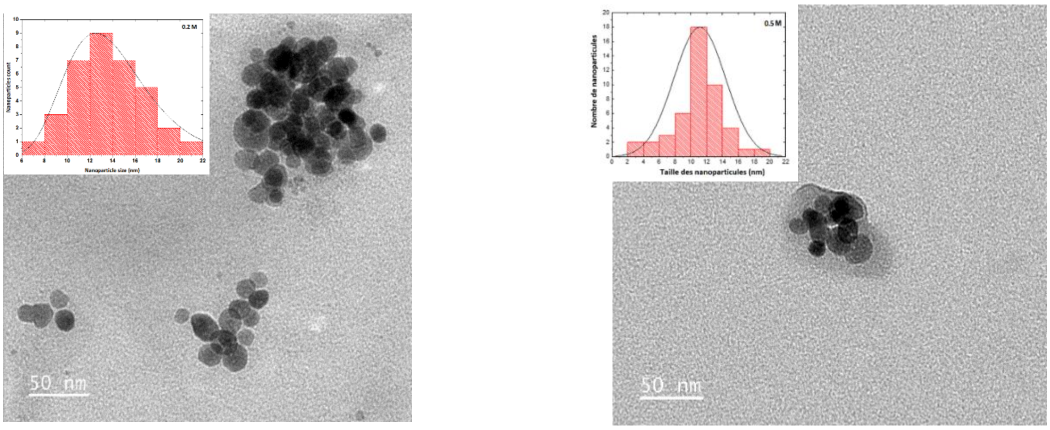

(a) (b)

Figure S3. TEM images of Fe_3O_4 nanoparticles synthesized by coprecipitation and coated by PA at (a) 0.2 and (b) 0.5 mol L^{-1} and. Inserts: Size distribution of individual nanoparticles with lognormal fit.

3.5.2. Transmission electron microscopy imaging of radiation-induced and AG-coated Fe_3O_4 nanoparticles

The mean diameter measured by TEM of Fe_3O_4 nanoparticles coated by Arabic gum (AG) and synthesized at 20 kGy is D = 3.9 nm and is smaller than at 60 kGy with D = 5.6 m (Figure S4).

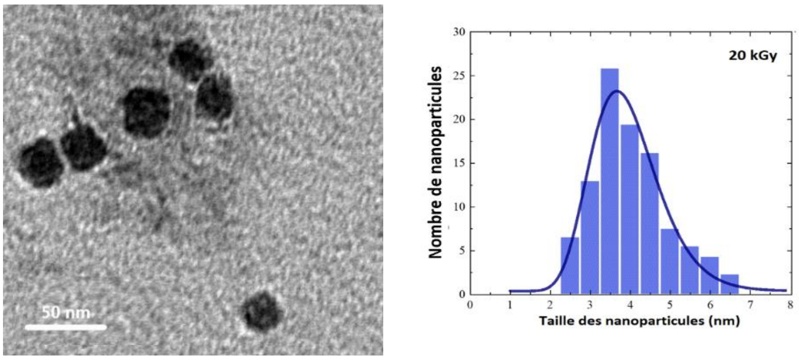

(a)

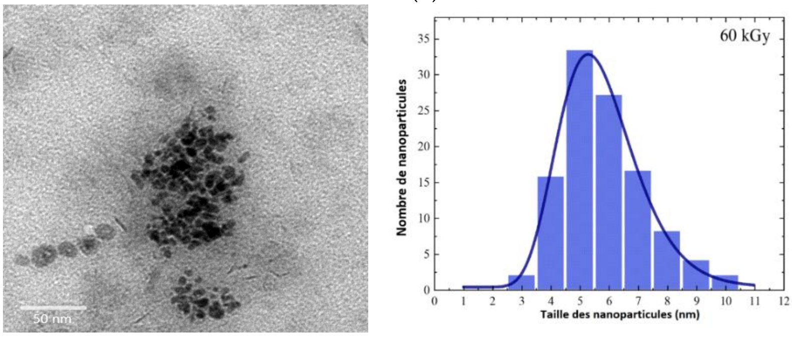

(b)

Figure S4. TEM images and diameter distribution with lognormal adjustment of Fe_3O_4 nanoparticles in the presence of arabic gum (AG), after an irradiation dose of: (a) 20 kGy; (b) 60 kGy.

3.6. Magnetic properties

3.6.1. Coprecipitated PA-coated Fe_3O_4 nanoparticles

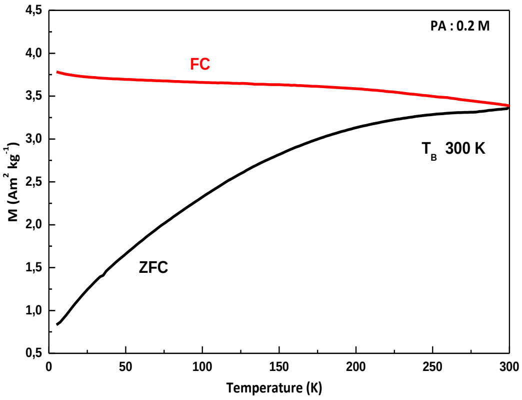

(a)

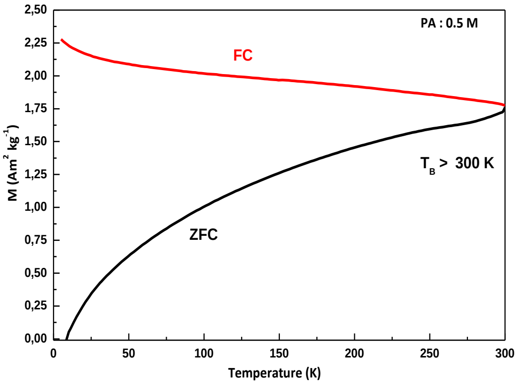

(b)

Figure S5. ZFC and FC magnetization curves for coprecipitated and PA-coated Fe_3O_4 nanoparticles at 0.2 and 0.5 mol L^{-1} (H = 50 Oe).

3.6.2. Radiation-induced Fe_3O_4 nanoparticles coated by AG

The magnetic properties of the ultra-small AG-coated Fe_3O_4 particles synthetized by irradiation at 60 kGy were investigated at 5 and 300 K as a function of external magnetic field (- 50 kOe < H < 50 kOe) (Figure S3 and Table S2).

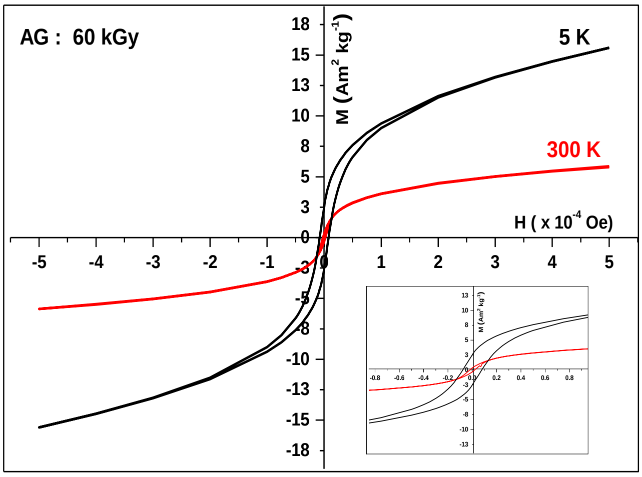

Figure S6. Magnetization loops at 5 and 300 K for Fe_3O_4 AG-coated nanoparticles synthetized at 60 kGy. Inset : Zoom at low field values.

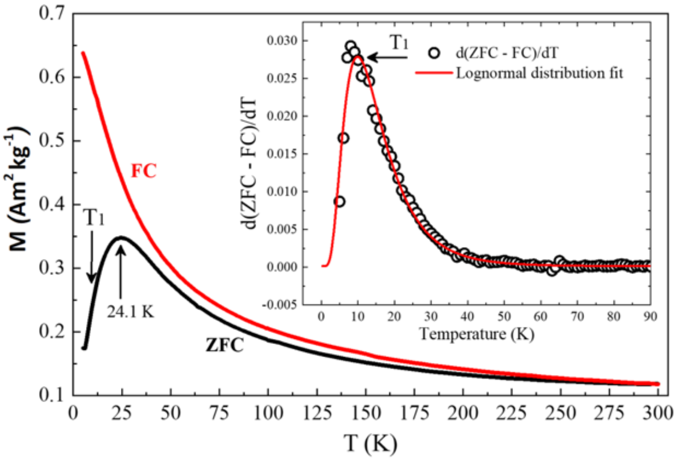

Figure S7. ZFC curve (in black) and FC curve (in red) of the magnetization of nanoparticles Fe_3O_4 radiation-induced (dose = 60 kGy) and AG-coated, H = 50 Oe. Insert: In red: lognormal adjustment of the derivative d(ZFC - FC)/dT.

Table S4. Magnetic properties of radiation-induced and AG-coated nanoparticles synthetized at 60 kGy.

	5 K			300 K		
**Dose (kGy)**	**M_r (A m^2 kg^{-1})**	**M_S (A m^2 kg^{-1})**	**H_C (Oe)**	**M_r (A m^2 kg^{-1})**	**M_S (A m^2 kg^{-1})**	**H_C (Oe)**
60	2.5	15.6	300	0.3	5.9	200

This evolution is probably due not only to the size distribution of the nanoparticles but also to the presence of other molecules such as AG molecules and NaCl crystals. According to the temperature-dependence of the magnetic properties, we conclude that these radiation-induced nanoparticles exhibit a superparamagnetic behaviour above T_B. However, the saturation magnetization at room temperature of radiation-induced Fe_3O_4 nanoparticles coated by AG Ms = 5.9 A m^2 kg^{-1} is found to be much lower than for radiation-induced ones coated by PA (Ms = 50.1 A m^2 kg^{-1}).
